# Brain Herniation into Giant Arachnoid Granulation: An Unusual Case

**DOI:** 10.1155/2017/8532074

**Published:** 2017-03-14

**Authors:** Joana Ruivo Rodrigues, Gonçalo Roque Santos

**Affiliations:** ^1^Radiology Unit, Medical Imaging Department, Centro Hospitalar de Tondela-Viseu, EPE, Viseu, Portugal; ^2^Neuroradiology Unit, Medical Imaging Department, Centro Hospitalar de Tondela-Viseu, EPE, Viseu, Portugal

## Abstract

Arachnoid granulations are structures filled with cerebrospinal fluid (CSF) that extend into the venous sinuses through openings in the dura mater and allow the drainage of CSF from subarachnoid space into venous system. Usually they are asymptomatic but can be symptomatic when large enough to cause sinus occlusion. We report a rare case of a brain herniation into a giant arachnoid granulation in an asymptomatic elderly male patient, which was discovered incidentally.

## 1. Introduction

Arachnoid granulations represent growths of arachnoid membrane into the dural sinuses through which CSF enters the venous system [1] and are macroscopically visible [2]. They range from a few millimetres to more than 1 cm (giant arachnoid granulations) [3] and consequently may grow to fill and dilate the dural sinuses or expand the inner table of the skull. Rarely, they cause symptoms related with venous hypertension secondary to partial sinus occlusion and usually they are incidental findings [1].

The presence of preexisting arachnoid granulations facilitates the formation of brain herniation into the dural venous sinus (DVS) or adjacent calvarium. The herniations of brain parenchyma into arachnoid granulations are thought to arise spontaneously or as a result of increased intracranial pressure [4].

## 2. Case Presentation

A 91-year-old man was admitted to our emergency department with an episode of sudden onset of confusion and right arm paraesthesia (without loss of consciousness), lasting less than 3 minutes. Upon admission, neurological exam was normal. Blood pressure was elevated (190/90 mmHg) and heart rate was stable (90 beats/minute). He had a history of type II Diabetes Mellitus, arterial hypertension, and a left hemicolectomy four years prior, in relation with colon adenocarcinoma (pT2N0M0).

**Figure 1:**
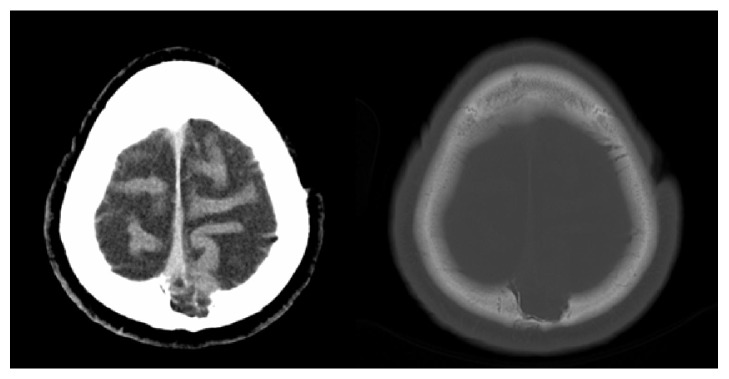
Head CT scan images show an incomplete bone defect in the left parietal bone, with heterogeneous density, including calcified areas.

**Figure 2:**
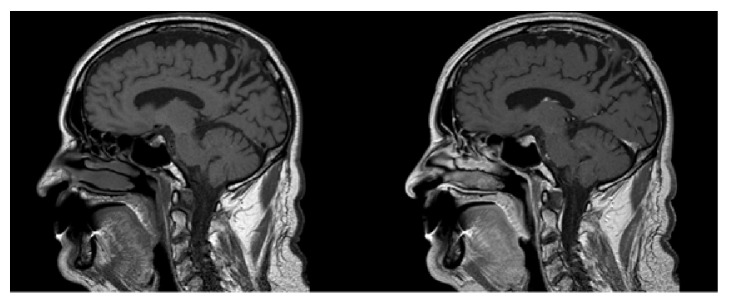
Sagittal T1-weighted images without and with contrast show brain herniation to the bone defect, without enhancement.

**Figure 3:**
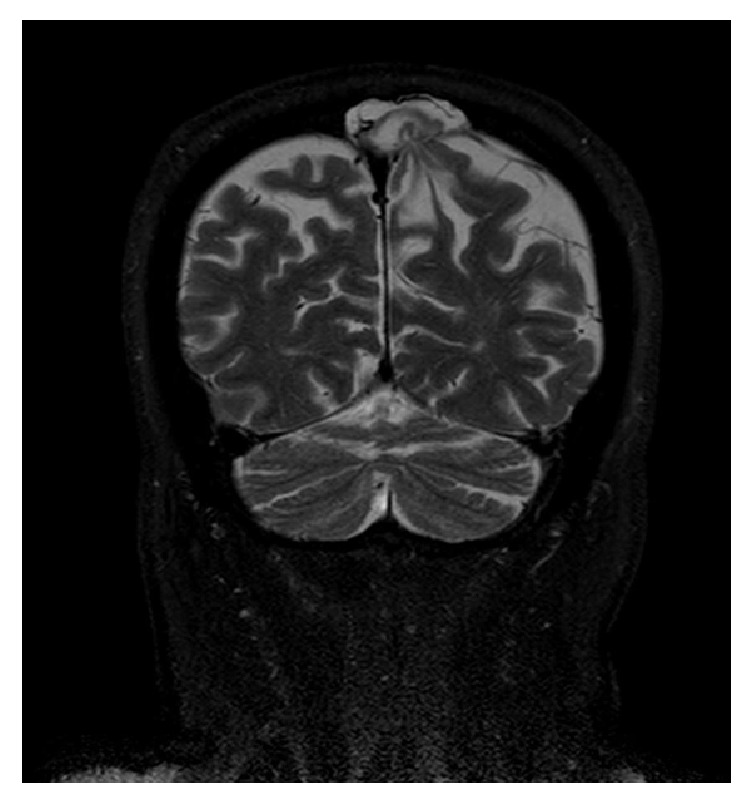
Coronal T2-weighted SPAIR image shows narrow neck and atrophy of the brain herniation.

**Figure 4:**
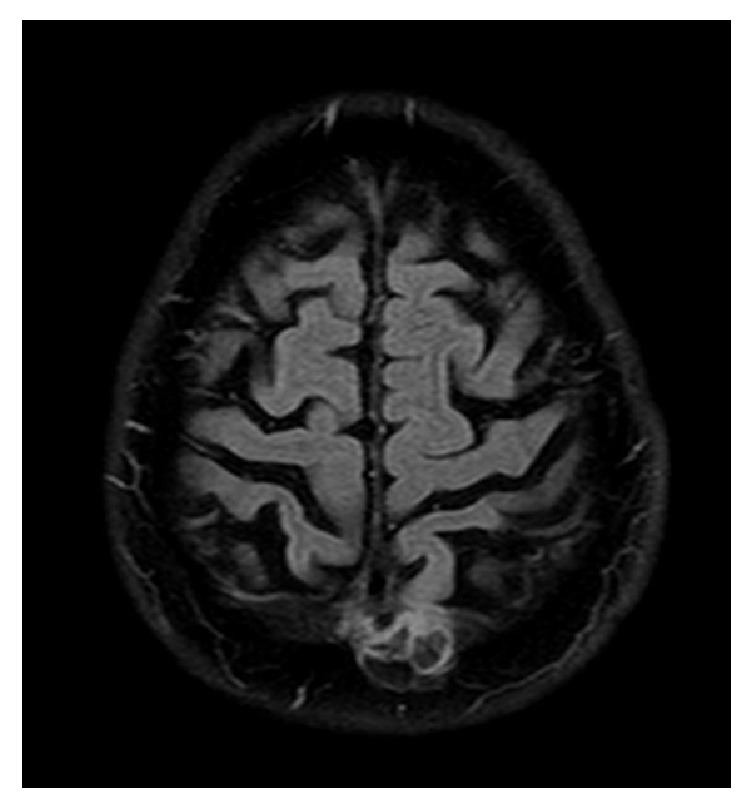
Axial T2-weighted FLAIR image shows hyperintensity of the brain herniation, probably due to prior infarction.

An urgent noncontrast computed tomography (CT) of the brain excluded acute vascular or posttraumatic lesions but showed an area of bone rarefaction localized in the parasagittal left parietal bone, associated with an ipsilateral parietal encephaloclastic cortical and subcortical brain lesion (Figure 1). Magnetic resonance imaging (MRI) of the brain was performed 2 months later (Figures 2, 3, and 4) and demonstrated an unenhancing brain herniation to the incomplete bone defect, with a narrow neck, atrophy, and hyperintensity on T2 FLAIR images. The incomplete bone defect was close to the superior sagittal sinus, posterior and superior to it.

These findings were found to be compatible with a brain herniation into a giant arachnoid granulation, with strangulation and infarction of the herniated brain tissue.

## 3. Discussion

Arachnoid granulations (AG) were first described by Antonio Pacchioni in 1705 [5]. They represent growths of arachnoid membrane into dural sinuses, through which CSF enters the venous system from subarachnoid space [6].

A large AG is called “giant” when larger than 1 cm [3]. However, Kan et al. referred to AG as “giant” when they fill the lumen of a dural sinus, causing local dilatation or filling defects [1].

AG can enlarge with age [7] or in response to an increase in cerebrospinal fluid pressure [8] and they can be found anywhere in the DVS [3]. Mostly giant AG are incidentally discovered on brain studies without any relation to the patients symptoms [1], as occurred in our case.

AG can appear on skull radiography as radiolucent zones or as impressions on the inner table of the calvaria. On CT AG can be hypodense or isodense relative to the brain parenchyma. On MR imaging, they are hyperintense on T2-weighted images and iso- or hypointense relative to brain parenchyma on T1-weighted images [3, 7]. Trimble et al. [3] reported that approximately 80% of giant arachnoid granulations contain CSF-incongruent fluid on at least one MR image and nearly half contain fluid that does not parallel CSF on at least two sequences. FLAIR is the most reliable technique, differing in signal intensity with CSF in 100% of cases [3]. The fluid in the granulations is almost not attenuated on a FLAIR sequence but remains hyperintense, most likely due to pulsation artifacts from the adjacent sinus and differing CSF flow characteristics within the AG [9].

On angiographic studies AG appear as ovoid filling defects in the dural venous sinuses in the venous phase [10]. The internal veins appear as focal linear contrast enhancement on enhanced CT or as linear flow voids on nonenhanced MR [10]. Nonvascular soft tissue can be present in giant AG, and it was interpreted as stromal collagenous tissue, hypertrophic arachnoid mesangial cell proliferation, or invaginating brain tissue [3]. However, it is possible that venous structures and/or connective tissues into the arachnoid granulations may be tamponed by mass effect from the herniated parenchyma, thereby making them invisible [4].

It is important to distinguish giant AG from pathologic processes in the dural venous sinuses, like thrombosis and neoplasia [10]. Thrombosis usually involves an entire segment of sinus or multiple sinuses and can extend into cortical veins, while AG produce focal, well-defined defects [6, 11]. The differential diagnosis with tumour can be made because of the shape, the lack of contrast enhancement, and the lack of diffusion restriction [10]. Brain parenchyma herniations into the calvarium are rare, recently described, and controversial in significance.

Brain parenchyma herniations with surrounding CSF into the DVS and/or calvarium have a prevalence of 0.32% and were encountered more frequently in posterior inferior parts of the intracranial cavity [12].

Malekzadehlashkariani et al. [13] retrospectively analyzed 38 patients with brain herniation into AG and found 68 brain herniations into AG, by order of frequency, in the occipital squama, transverse sinus, lateral lacuna of the superior sagittal sinus, and straight sinus, with cerebellar tissue being the most frequently found in the herniation. Brain parenchyma herniations affect more women than men [13].

Chan et al. [14] described a focal brain herniation into a giant arachnoid granulation located in a DVS which may have developed spontaneously or was induced by elevated intracranial pressure and cerebral edema resulting from prior head trauma.

Çoban et al. [15] reported a symptomatic brain herniation as an occult temporal lobe encephalocele into a transverse sinus. They coined the term “occult encephalocele” due to its distinguishing features from those of more common encephaloceles.

Battal and Castillo [4] observed five patients showing brain herniation into the DVS or calvarium containing various amounts of cerebral or cerebellar parenchyma surrounded by CSF. Battal and Castillo [4] concluded that herniations of brain parenchyma into arachnoid granulations can occur spontaneously or as a result of increased intracranial pressure. Battal et al. [12] in another study referred that the herniations were incidentally detected in all patients and the majority occurred spontaneously, but few were associated with masses that presumably could have increased intracranial pressure. It was suggested that the underlying giant arachnoid granulation might be a predisposing factor.

Brain herniations are best detected with high-resolution T1- and T2-weighted MRI sequences and higher field strengths [12].

Battal et al. [16] believe that brain herniations into the skull have different features from classic encephaloceles, since the brain herniations that were described by the previous referred authors did not occur through a complete calvarial defect but instead occurred through a dural defect into calvarium. Those cases did not show external bony defects that are usually present in encephaloceles.

Brain herniations into the calvarium are very rare, their clinical significance is not well-known, and they should be considered in the differential diagnosis of encephaloceles. The continuity of the external table of the calvarium can be the distinguishing feature of this entity from encephalocele. The presence of preexisting arachnoid granulations facilitates the formation of brain herniation into the DVS or adjacent calvarium [4, 16].

Our case demonstrated such unusual complication of brain herniation into a giant arachnoid granulation, in a rare and unique location: the convexity area (left parasagittal parietal region). Those findings were discovered incidentally without any relation to the patient symptoms. Strangulation (in probable association with a narrow neck) and infarction are present in the herniated parenchyma. Future potential complications, such as brain dysfunction and seizures, should not be ignored. Therefore accurate diagnosis is required and care must be taken not to mistake for a neoplasm. Prompt treatment should be directed at the underlying cause, when it is known.

In conclusion, we have presented a rare and unique case of brain herniation in the convexity area into a giant arachnoid granulation, without any history of trauma or of elevated intracranial pressure.

## References

[1] Kan P., Stevens E. A., Couldwell W. T. (2006). Incidental giant arachnoid granulation. *American Journal of Neuroradiology*.

[2] Potts D. G., Reilly K. F., Deonarine V. (1972). Morphology of the arachnoid villi and granulations. *Radiology*.

[3] Trimble C. R., Harnsberger H. R., Castillo M., Brant-Zawadzki M., Osborn A. G. (2010). “Giant” arachnoid granulations just like CSF?: NOT!!. *American Journal of Neuroradiology*.

[4] Battal B., Castillo M. (2014). Brain herniations into the dural venous sinuses or calvarium: MRI of a recently recognized entity. *Neuroradiology Journal*.

[5] Brunori A., Vagnozzi R., Giuffrè R. (1993). Antonio Pacchioni (1665–1726): early studies of the dura mater. *Journal of Neurosurgery*.

[6] Chin S. C., Chen C. Y., Lee C. C. (1998). Giant arachnoid granulation mimicking dural sinus thrombosis in a boy with headache: MRI. *Neuroradiology*.

[7] Leach J. L., Jones B. V., Tomsick T. A., Stewart C. A., Balko M. G. (1996). Normal appearance of arachnoid granulations on contrast-enhanced CT and MR of the brain: differentiation from dural sinus disease. *American Journal of Neuroradiology*.

[8] Grossman C. B., Potts D. G. (1974). Arachnoid granulations: radiology and anatomy. *Radiology*.

[9] Leach J. L., Meyer K., Jones B. V., Tomsick T. A. (2008). Large arachnoid granulations involving the dorsal superior sagittal sinus: findings on MR imaging and MR venography. *American Journal of Neuroradiology*.

[10] De Keyzer B., Bamps S., Van Calenbergh F., Demaerel P., Wilms G. (2014). Giant arachnoid granulations mimicking pathology: a report of three cases. *Neuroradiology Journal*.

[11] Choi H. J., Cho C. W., Kim Y. S., Cha J. H. (2008). Giant arachnoid granulation misdiagnosed as transverse sinus thrombosis. *Journal of Korean Neurosurgical Society*.

[12] Battal B., Hamcan S., Akgun V. (2016). Brain herniations into the dural venous sinus or calvarium: MRI findings, possible causes and clinical significance. *European Radiology*.

[13] Malekzadehlashkariani S., Wanke I., Rüfenacht D. A., San Millán D. (2016). Brain herniations into arachnoid granulations: about 68 cases in 38 patients and review of the literature. *Neuroradiology*.

[14] Chan W. C., Lai V., Wong Y. C., Poon W. L. (2011). Focal brain herniation into giant arachnoid granulation: a rare occurrence. *European Journal of Radiology Extra*.

[15] Çoban G., Yildirim E., Horasanli B., Çifçi B. E., Ağıldere M. (2013). Unusual cause of dizziness: occult temporal lobe encephalocele into transverse sinus. *Clinical Neurology and Neurosurgery*.

[16] Battal B., Hamcan S., Akgun V., Sari S., Karaman B. (2015). Brain herniation with surrounding CSF into the skull or encepholecele?. *Journal of Neuroradiology*.

